# A secular trend in age at menarche in Yunnan Province, China: a multiethnic population study of 1,275,000 women

**DOI:** 10.1186/s12889-021-11951-x

**Published:** 2021-10-19

**Authors:** Wen Liu, Xuejing Yan, Chengyu Li, Qi Shu, Meng Chen, Le Cai, Dingyun You

**Affiliations:** 1 Department of Thyroid Surgery, the First Affiliated Hospital of Kunming Medical University, Kunming, 650032 China; 2 Department of Management of Chronic Non-communicable Diseases, Yunnan Center for Diseases Control and Prevention, Kunming, 650032 China; 3 School of Public Health, Kunming Medical University, Kunming, 650500 China; 4 No. 1 School of Clinical Medicine, Kunming Medical University, Kunming, 650032 China

**Keywords:** Birth cohort, Menarche, Menarche age, Secular trend, Multiethnic, Population study

## Abstract

**Background:**

Age at menarche (AAM) has shown different trends in women from different ethnic and economic regions in recent decades. Data on AAM among multiethnic women living in developing areas are scarce.

**Methods:**

Data on AAM from 1,275,000 women among 26 ethnicities in Yunnan Province, China, who were born from 1965 to 2001 were obtained from the National Free Preconception Health Examination Project from 2010 to 2018. The patterns of AAM trends were analysed according to ethnic group, area of residence, and socioeconomic status.

**Results:**

The mean AAM was 13.7 ± 1.21 years (95% CI 13.697–13.701), with a decrease from 14.12 (±1.41) among women born before 1970 to 13.3 (±1.04) among those born after 2000. The decline was 0.36 years per 10-year birth cohort, and the plateau has not yet been reached in Yunnan. A secular trend of earlier AAM was observed in all 26 ethnic groups. The fastest rate of decline was observed for the Bai ethnicity (0.36 years per decade). Consistent declining trends in AAM appeared among extreme-, middling-, and nonpoverty economic patterns from 1965 to 2001, with reductions of 1.19, 1.44, and 1.5 years, respectively (*P* < 0.001). The peak reduction among middling poverty and extreme poverty occurred in the early 2000s (0.4 and 0.32 years). Multivariate analysis showed a significant difference in the declining trends in AAM along rural/urban lines (*P* < 0.001).

**Conclusion:**

There was a secular trend towards a younger AAM during the twentieth century and early twenty-first century birth cohorts in the Yunnan population. Considering the difference in AAM trends due to ethnic and socioeconomic status in Yunnan, the health authority should utilize flexible adjusted health care strategies in different regions.

**Supplementary Information:**

The online version contains supplementary material available at 10.1186/s12889-021-11951-x.

## Background

Age at menarche (AAM) is one of the markers that indicates the onset of puberty in girls. As an evolutionary adaptive mechanism, AAM balances early reproduction, large body size, fertility, and potential mortality risk in pregnancy [1]. AAM can be affected by genetic, economic, nutritional, and social environmental factors [2–4]. One study showed a secular trend towards earlier AAM in Europe and the United States (US), with a decrease from 15 to 16 years to 12–13 years during the nineteenth century to the middle of the twentieth century [5]. Several recent studies have suggested that the trend may be related to economic growth and social environment changes because these can directly benefit nutrition and health care [6–8]. Compared to 1955–1965, the trend for early AAM in Dutch women slowed down in 1965–1997 (3 months vs. less than 1 month per decade), while Netherland’s gross domestic product (GDP) grew by almost 20 times in later decades [9]. This result suggested that there was no linear relationship between economic growth and AAM, instead of remaining relatively stable when the threshold was reached. In fact, there are two different trends for AAM including continuous reduction and levelling off in different countries and regions [10–13].

Earlier AAM is an important indicator not only for sexual development but also for some diseases and social problems, such as obesity, diabetes, cardiovascular disease and adolescent psychological problems [14–19]. Therefore, it is critical to survey AAM with a regionally inclusive and large population sample to protect women’s health, and the information obtained can be used to adjust health management programmes, such as disease screening and intervention measures for the target age groups. Compared to European studies over two centuries on AAM, however, efforts are still scarce in certain disadvantaged areas of Asia and Africa. An investigation among students of 13 ethnic minorities showed that the median AAM ranged from 12.1 to 13.6 years in Southwest China in 2014 [20]. However, these data might not reflect the real AAM trend in some poverty-stricken areas, as Yunnan Province includes vast territory, a large population, and many diverse ethnicities.

There are 26 diverse ethnicities living in Yunnan Province, which is the most ethnically diverse area in China. Yunnan had approximately 47.2 million permanent residents and a GDP per capita of approximately 7977 dollars in 2020, while the GDP per capita was only approximately 314, 136, and 962 dollars in 1980, 1990 and 2000, respectively [21]. The economy continues to grow. Because early AAM is associated with women’s health problems in later adulthood, the analysis of AAM trends is useful for health authorities to update women’s health policies. Therefore, the investigation of AAM in this region has high reference value for less developed countries/regions and homogeneous ethnic regions (e.g., South Asia and Southeast Asia).

In this study, we aimed to investigate the trend in AAM over the twentieth century and early twenty-first century within a multiethnic large population study from Yunnan Province, China, hypothesizing that AAM has declined over time, in accordance with global trends. We also aimed to describe the patterns, magnitudes and rates of the change in AAM between groups defined by ethnic and socioeconomic states, and potential health risks in women were assessed.

## Methods

### Study design and recruitment

This study was a part of the National Free Preconception Health Examination Project (NFPHEP), which was launched by the National Health and Family Planning Commission and the Ministry of Finance of China in 2010. The project provided free preconception examinations in rural areas to fertile women who planned a pregnancy within the next 6 months. Detailed information on the project was described in previous reports [22, 23].

In our retrospective cohort, 1,293,028 women were recruited to join the NFPHEP from 1st January 2010 to 31st December 2018. It covered all areas in Yunnan Province, China (129 counties/districts in 16 cities).

### Data collection

Data collection for this project was based on the primary health and family planning network in China. Baseline information (age, ethnicity, and address of residence) of participants and their husbands was collected during face-to-face interviews by trained and certified interviewers with a structured questionnaire. In the questionnaire, the participants were asked how old (in years) they were at the age of menarche. A total of 18,028 women whose questionnaires did not meet the integrality criterion were excluded.

### Variables

Recalled AAM served as the dependent variable, according to year of birth (1965–2001), for the group participants’ birth cohorts (<1970s, 1970s, 1980s, 1990s, and early 2000s). Ethnic information was collected from individual household registration. According to the traditional definition, ethnic groups other than Han are ethnic minorities, accounting for less than 10% of China’s population. The socioeconomic status of participants was determined through the area of residence (rural and urban) and the degree of poverty in their regions (extreme poverty, middling poverty, and nonpoverty) as defined by the State Council Leading Group Office of Poverty Alleviation and Development. The standard of economic indicators for middling poverty was less than 211.5 dollars per year for GDP per capita in 1992. If large-scale regional poverty was sustained for more than 5 years (less than the dynamic standard, e.g., 528.9 dollars in 2020), then the corresponding regions were defined as experiencing extreme poverty [24, 25]. All data reflect the status of respondents at the time of interview.

### Statistical analyses

All statistical analyses were performed using R4.0.2. For continuous variables, T test and ANOVA test were applied to calculate the mean AAM across birth cohorts, ethnicity, area of residence, and economic status. The linear regression was used in the comparison for the trends of different stratified subgroups (e.g. ethnicity, urban/rural, or economic group). Graphs were drawn by the ‘ggplot2’ package.

## Results

**Fig. 1:**
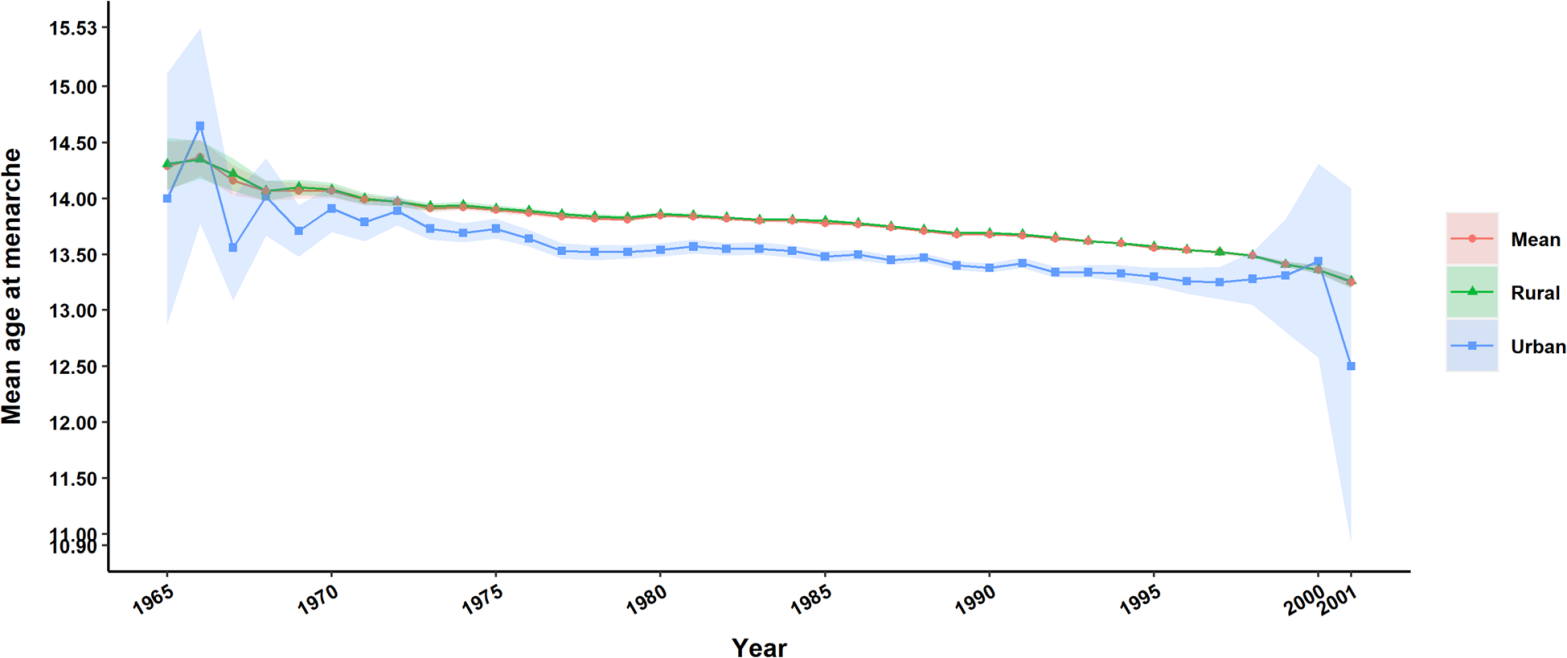
Secular trend of age at menarche in Yunnan Province, China

Overall, 1,275,000 women met the inclusion criteria for this survey. The mean age was 27.03 ± 5.61 years, and the mean AAM was 13.7 ± 1.21 years (95% confidence interval [CI] 13.697–13.701). Our study has shown a secular trend towards an earlier AAM in Yunnan during the twentieth century and early twenty-first century, with large amounts of data showing a decrease from 14.12 (±1.41) for women born before 1970 to 13.3 (±1.04) for those born after 2000. There was a reduction of 1.35 years (16.2 months) over an interval of 38 years (from 1965 to 2001) (*P* < 0.001), with the decline occurring at 0.36 years (4.3 months) per 10-year birth cohort by linear regression (*P* < 0.001) (Fig. 1) The mean AAM data across birth cohorts, ethnicity, area of residence, and economic status among Yunnan women are shown in Table 1 and Supplement 1.

**Table 1:** Mean Age At Menarche across birth cohorts, ethnicity, area of residence, and economic status among Yunnan women during the study period

		Age at menarche		
	Total (%) *N* = 1,275,000	Mean (SD)	95% CI	*P* value
**Birth cohort**^a^				<0.0001
<1970s	3380(0.261)	14.119(1.405)	14.072–14.166	
1970s	109,206(8.446)	13.866(1.283)	13.859–13.874	
1980s	575,938(44.542)	13.755(1.233)	13.752–13.759	
1990s	575,103(44.477)	13.613(1.170)	13.610–13.616	
Early 2000s	4985(0.386)	13.296(1.039)	13.267–13.325	
**Ethnic group**^b^				<0.0001
Han	814,111(63.852)	13.671(1.179)	13.669–13.674	
Yi	152,381(11.951)	13.772(1.281)	13.765–13.778	
Dai	46,448(3.643)	13.610(1.237)	13.599–13.621	
Bai	38,134(2.990)	13.842(1.329)	13.829–13.856	
Miao	38,420(3.013)	13.655(1.141)	13.644–13.666	
Hani	37,022(2.904)	13.792(1.291)	13.779–13.805	
Zhuang	32,857(2.577)	13.665(1.235)	13.652–13.679	
Lisu	24,760(1.942)	14.058(1.369)	14.041–14.075	
Hui	15,487(1.215)	13.403(1.099)	13.386–13.420	
Laku	13,802(1.183)	13.721(1.220)	13.701–13.741	
Wa	12,679(0.994)	13.631(1.103)	13.611–13.650	
The others^c^	41,106(3.224)	13.865(1.307)	13.853–13.878	
**Area of residence**^d^				<0.0001
Rural	1,231,899(96.620)	13.707(1.212)	13.705–13.709	
urban	43,094(3.380)	13.477(1.181)	13.466–13.488	
**Economic status**				<0.0001
Extreme poverty	343,014(26.903)	13.799(1.142)	13.795–13.803	
Middling poverty	549,681(43.112)	13.711(1.235)	13.708–13.714	
Non-poverty	382,305(29.985)	13.593(1.231)	13.589–13.597	

^a^ Data regarding the birth cohort were missing for 6388 cases (0.5%)

^b^Data regarding the ethnicity were missing for 7793 cases (0.6%)

^c^See Supplement 1 for detail

^d^Data regarding the area of residence were missing for 7 cases

### Age at menarche in ethnic groups

An overall consistent secular trend of earlier AAM is shown in all ethnic groups described in Table 2 and Supplement 1. However, the rates of decline have exhibited heterogeneity in different ethnic groups. Faster rates of decline were found in the Bai ethnicity (4.3 months per decade); a slower rate of decline was seen in the Lisu ethnicity (1.9 months per decade). The decline in the Han population, which has the largest population, was 2.5 months per decade.

**Table 2:** A secular trends in age at menarche among 1,275,000 women in Yunnan province according to birth cohorts

	Age at Menarche – years±SD (95%CI)^a^						*P* for trend
	<1970s	1970s	1980s	1990s	Early 2000s	Difference	
**Ethnic group**							
Han	14.085 ± 1.361 (14.029–14.142)	13.820 ± 1.240 (13.811–13.828)	13.715 ± 1.19 4(13.711–13.719)	13.595 ± 1.144 (13.592–13.599)	13.298 ± 1.039 (13.256–13.340)	(− 0.7876)(− 0.8574,-0.7178)	<0.0001
Yi	14.180 ± 1.498 (14.008–14.352)	13.939 ± 1.368 (13.914–13.964)	13.834 ± 1.303 (13.824–13.843)	13.678 ± 1.234 (13.669–13.688)	13.408 ± 1.159 (13.305–13.511)	(− 0.7725)(− 0.9604,-0.5846)	<0.0001
Dai	14.019 ± 1.352 (13.646–14.391)	13.813 ± 1.302 (13.763–13.864)	13.655 ± 1.250 (13.639–13.672)	13.544 ± 1.211 (13.527–13.560)	12.979 ± 1.143 (12.791–13.168)	(− 1.0397)(− 1.4207,-0.6587)	<0.0001
Bai	14.413 ± 1.696 (14.166–14.660)	14.029 ± 1.366 (13.986–14.071)	13.898 ± 1.342 (13.879–13.917)	13.711 ± 1.280 (13.690–13.732)	13.053 ± 1.038 (12.711–13.394)	(− 1.3604)(− 1.9239,-0.7969)	<0.0001
Miao	14.202 ± 1.471 (13.901–14.503)	13.962 ± 1.275 (13.909–14.016)	13.802 ± 1.182 (13.782–13.822)	13.552 ± 1.091 (13.538–13.567)	13.280 ± 0.946 (13.222–13.339)	(− 0.9218)(− 1.1336,-0.71)	<0.0001
Hani	14.329 ± 1.527 (14.079–14.579)	14.152 ± 1.456 (14.103–14.201)	13.950 ± 1.353 (13.929–13.970)	13.563 ± 1.14 1(13.545–13.580)	13.255 ± 1.087 (13.049–13.460)	(− 1.0742)(− 1.4114,-0.737)	<0.0001
Zhuang	14.000 ± 1.449 (13.680–14.320)	13.868 ± 1.358 (13.816–13.920)	13.732 ± 1.263才 (13.711–13.754)	13.576 ± 1.181 (13.558–13.594)	13.211 ± 1.013 (13.069–13.353)	(− 0.7889)(− 1.0887,-0.4892)	<0.0001
Lisu	14.119 ± 1.274 (13.809–14.430)	14.224 ± 1.521 (14.153–14.296)	14.158 ± 1.409 (14.130–14.186)	13.971 ± 1.313 (13.948–13.993)	13.532 ± 0.958 (13.395–13.669)	(− 0.5878)(− 0.8812,-0.2944)	0.0001
Hui	13.885 ± 1.451 (13.298–14.471)	13.583 ± 1.169 (13.506–13.661)	13.456 ± 1.117 (13.427–13.486)	13.353 ± 1.080 (13.331–13.376)	13.000 ± 0.977 (12.706–13.294)	(− 0.8846)(− 1.4602,-0.309)	0.0031
Laku	14.000 ± 1.291(13.570–14.430)	13.968 ± 1.280 (13.887–14.050)	13.824 ± 1.237 (13.792–13.857)	13.616 ± 1.186 (13.588–13.644)	13.179 ± 1.111 (12.976–13.383)	(−0.8205)(−1.2514,-0.3896)	0.0002
Wa	13.692 ± 1.104c (13.334–14.050)	13.720 ± 1.041 (13.658–13.783)	13.651 ± 1.090 (13.623–13.679)	13.595 ± 1.127 (13.565–13.624)	12.979 ± 1.053 (12.670–13.288)	(−0.7136)(−1.1771,-0.25)	0.003
The others^b^	14.213 ± 1.473 (13.874–14.552)	14.085 ± 1.394c (14.036–14.135)	13.929 ± 1.333 (13.910–13.949)	13.770 ± 1.257 (13.752–13.789)	13.389 ± 1.024c (13.252–13.526)	(−0.8244)(−1.1293,-0.5196)	<0.0001
**Area of residence**							
Rural	14.141 ± 1.404 (14.092–14.191)	13.884 ± 1.286 (13.876–13.892)	13.767 ± 1.234 (13.763–13.770)	13.619 ± 1.169 (13.616–13.622)	13.296 ± 1.038c (13.267–13.325)	(−0.845)(− 0.8984,-0.7917)	<0.0001
urban	13.841 ± 1.391 (13.669–14.014)	13.621 ± 1.215 (13.594–13.649)	13.488 ± 1.175 (13.473–13.503)	13.361 ± 1.154 (13.341–13.382)	13.154 ± 1.14 4(12.463–13.845)	(−0.6874)(−1.4606,0.0858)	0.0812
**Economic status**							
Extreme poverty	14.155 ± 1.327c (14.072–14.238)	14.008 ± 1.259 (13.993–14.023)	13.870 ± 1.163 (13.864–13.876)	13.712 ± 1.196 (13.706–13.717)	13.391 ± 1.034 (13.346–13.435)	(−0.7645)(− 0.8507,-0.6783)	<0.0001
Middling poverty	14.178 ± 1.456 (14.103–14.252)	13.906 ± 1.316c (13.894–13.918)	13.784 ± 1.252 (13.779–13.789)	13.605 ± 1.190 (13.600–13.610)	13.215 ± 1.023 (13.174–13.256)	(−0.9627)(−1.0416,-0.8837)	<0.0001
Non-poverty	13.990 ± 1.398 (13.901–14.080)	13.711 ± 1.240 (13.698–13.724)	13.631 ± 1.247 (13.626–13.637)	13.519 ± 1.205c (13.513–13.525)	13.285 ± 1.092c (13.195–13.376)	(−0.7052)(− 0.8405,-0.57)	<0.0001

^a^ Data regarding the age at menarche of birth cohort were missing for 6388 cases (0.5%)

^b^ See Supplement 1 for detail

### Age at menarche by socioeconomic status

**Fig. 2:**
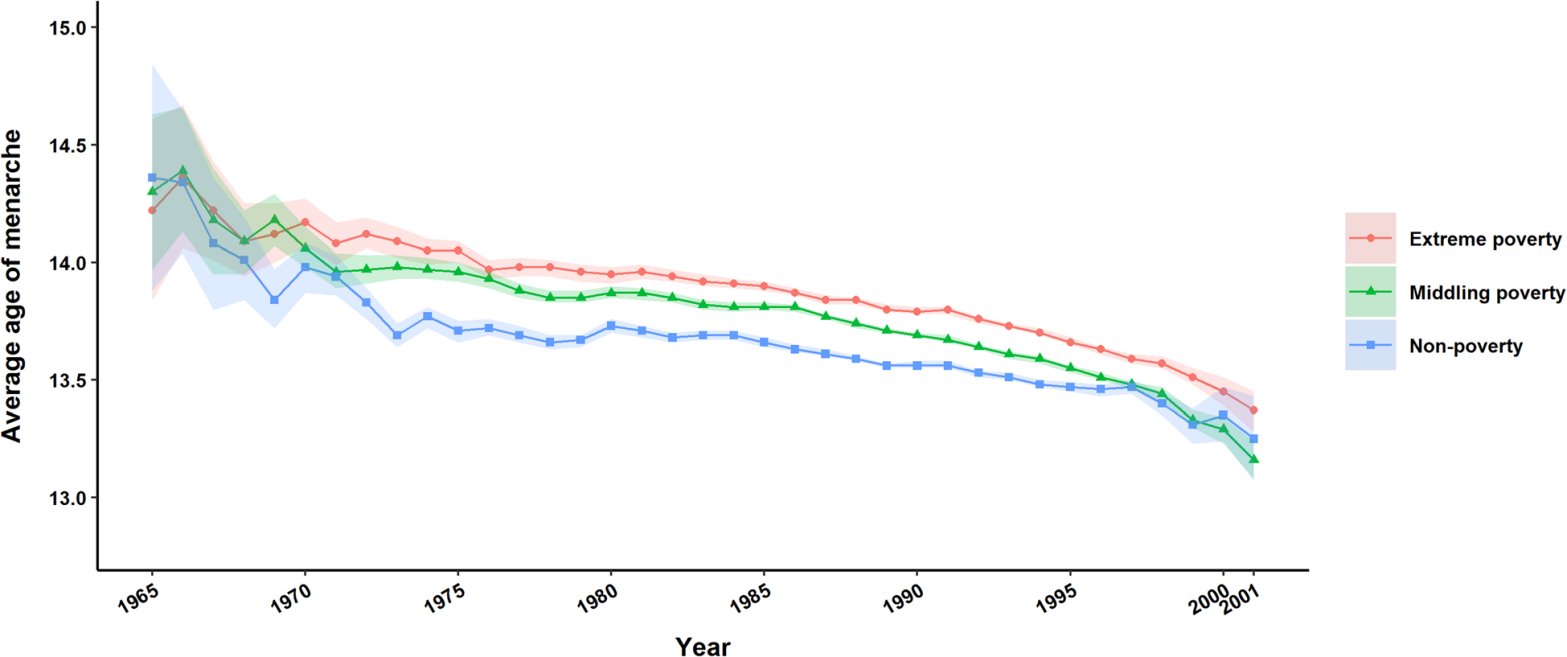
Secular trend in age at menarche in Yunnan Province by area of residence

The socioeconomic effect of early AAM seemed to be continuously present in Yunnan across birth cohorts. In terms of patterns, the decline in AAM for poverty groups was relatively faster, with the peak of reduction among middle poverty and extreme poverty occurring in the early 2000s (0.4 and 0.32 years, respectively). In contrast, the decline of nonpoverty women was more gradual, with a peak for the 1970s cohort (0.28 years). Overall, the declining trends in AAM appeared among the three economic status patterns from 1965 to 2001, with reductions of 1.19, 1.44, and 1.5 years for extreme-, middling-, and nonpoverty, respectively (*P* < 0.001). The difference between the populations in extreme poverty and nonpoverty was on average 0.21 years (2.5 months) across birth cohorts, but that gap decreased significantly from 0.3 years (3.6 months) in the 1970s birth cohort to 0.1 years (1.2 months) in the early 2000s counterpart cohort. We also found that the gradual reduction in the AAM gap along rural/urban lines did not exhibit a distinct difference (from 0.3–0.26 years in the <1970s - 1990s birth cohort to 0.15 years in the early 2000s). Multivariate analysis showed a significant difference in the declining trends in AAM between different household registration statuses (*P* < 0.001). However, we noted that selective bias might be unavoidable in only 13 cases in the early 2000s birth cohort for the urban group (Table 2, Figs. 1 and 2).

## Discussion

China’s large medical encyclopaedias recorded that the AAM of Chinese females was 14 years during the Tang dynasty period (618–906 AD) [26, 27]. In the second half of the twentieth century and early twenty-first century, AAM decreased to 12–13 years in most developed countries with the improvement of living conditions [28–32]. Recent Chinese pubertal surveys have shown that the median AAM values of schoolgirls were 12.11 and 12.10 years in two metropolises (Shanghai and Shenzhen), respectively, levels similar to developed countries [33, 34]. However, our study showed that the AAM of Yunnan women (13.7 ± 1.21) appeared much later than in those developed regions during the study period. There was a reduction of 1.35 years (16.2 months) over an interval of 38 years (from 1965 to 2001), and the AAM was 14.12 ± 1.41 for women born before 1970 and 13.3 ± 1.04 for those born after 2000. A secular trend towards earlier AAM was found in our study, suggesting that Yunnan could belong among the regions where AAM has not yet slowed down or stabilized. Simultaneously, our results show the decline in AAM, which exhibits important variations with respect to patterns, magnitudes and rates within different ethnic and socioeconomic populations and according to health risks.

### Ethnic groups

An American cohort study found that AAM before 12 years was more common in black girls than in their white counterparts (46% vs. 26%, *P* < 0.001) [2]. Across birth cohorts in Mexico over the twentieth century, nonindigenous residents on average reached the AAM earlier than their indigenous counterparts for both urban and rural women [4]. Towne et al. [35] confirmed that approximately 50% of the variation (heritability 0.49) in menstrual timing can be attributed to genetic factors that were also found to be associated with pubertal development parameters, such as weight and height in adolescents [36]. More than 450,000 ethnic minority women were included in the present study, and the proportion of Han ethnic and minority ethnic groups was approximately 2:1. Compared with Han ethnicity, the AAM of most minority women is different from that of Han ethnic women by approximately 3 months. This may be attributed to these ethnic groups; Han residents have been mixed for a long time, and living habits and cultural traditions have tended to assimilate. In our survey, women of the Lisu ethnicity were the group with the latest mean AAM (14.06 ± 1.37 years) and the least decreased magnitude (− 0.5878 years), respectively 4.7 months later and 2.4 months less than those of the Han ethnicity. These women are mainly distributed in southwest China, northern and central Burma, and the mountains of northern Thailand. Whether in China or Thailand, education, socioeconomic development, and health care are generally at low levels [37]. This suggests that it may be necessary to augment investment in health services in heavily populated Lisu ethnic areas. The earliest mean AAM group was Hui ethnic women, who experienced AAM 3.4 months earlier than Han ethnic women. Individuals of the Hui ethnicity are the most widely distributed ethnic minority group in China. Apart from mixed living with the Han ethnic group, beef and mutton intake in the Hui ethnic group is much greater than in other ethnicities because they adhere to Islamic customs and pork is forbidden. These animal proteins may cause earlier menarche by stimulating the release of insulin-like growth factor-1, which can regulate human growth and development [38, 39]. This finding suggests that the Hui ethnicity may need to be given priority in adolescent reproductive health education.

In fact, it is difficult to precisely distinguish the influence of either genetic or environmental factors. For example, earlier puberty was associated with earlier maternal puberty, but environmental changes usually occur simultaneously between family members. Additionally, some studies also observed earlier AAM among people who migrated from less to more economically developed countries [11]. This observation seemed to reflect environmental changes rather than ethnic factors.

### Women of different socioeconomic statuses encounter varying degrees of risk

Social and economic status inequalities remain prominent in developing countries and regions; they might be responsible for important shifts in AAM within and among countries [40]. In developed regions, the declining trend seems to slow down or level off after AAM reaches a certain threshold [26], such as among French (from 12.78 to 12.6 years among girls born in 1979–1994) [28], American (from 12.5 to 12.3 years among girls born in 1939–1993) [32], Dutch (13.15 years in 1997 to 13.05 years in 2009) [31], and Greek women (from 12.27 years in 1996 to 12.29 years in 2006) [30]. The AAM in Yunnan women was obviously later than that of the women of America [41–43], Finland [44], Sweden [45, 46], Denmark [47], the United Kingdom [48], Germany [49], the Netherlands [50], Belgium [51], Switzerland [52], Italy [53], Spain [54], and Greece [55] at the same time during our study period. Compared to the well-off populations in some Asian regions, such as China [56], Japan [57], India [58], and Thailand [59], in the same period, the lag time in AAM of Yunnan women appeared to be more evident. This result indicated that the downward trend of AAM in Yunnan may be likely to continue.

With economic growth and social environment changes, improvements in women’s nutritional status may be the main reason for earlier puberty. Good nutritional status, especially adequate intake of protein, can improve the function of the pituitary and thyroid glands and increase the secretion of growth hormone and thyroxine, with the age of growth subsequently shifted earlier. The AAM of Polish women showed an earlier trend with the rapid development of society and the economy from 1966 to 1978, whereas the AAM was delayed during the next decade of political turmoil [8].

Our findings echo previous studies with respect to significant differences in socioeconomic status: the average AAM in nonpoverty areas was 0.21 years ahead of that in areas with extreme poverty. A closer inspection by economic status and area of residence across birth cohorts reveals important variation within the Yunnan population in magnitude and rates of secular decline. The secular decline in AAM of the poverty and rural populations was more apparent than that of the nonpoverty and urban populations, with the gap between groups decreasing significantly (Figs. 1 and 2). The average AAM for women born into extreme poverty in the 1980s is significantly older than that of their nonpoverty counterparts in the same cohort. With the implementation of the Chinese government’s poverty alleviation policy, health services, nutritional status, social security and living conditions were continuously improving. These changes were reflected in the trend of a rapid decrease in AAM among women living in poverty and rural areas. From the public health perspective, however, the influence of early menarche age on women’s health should be given more attention by health management departments.

### Earlier age at menarche and women’s health risks

Many Mendelian randomization studies have shown a potential effect of earlier AAM on a broad range of health-related traits [60–63]. AAM less than 12 years can be considered as an indicator in adolescent health interventions [64]. Given the robust correlation between early AAM and women’s health risks, the trend of AAM in the previous study provides an alert to health authorities, and women’s health management should be adjusted to respond to the related disease threat.

As the improvements in education and healthcare level often lag behind the economy in impoverished areas, the declining trend in AAM reflected the needs of an increasingly younger youth group in Yunnan Province. Adolescent girls with earlier AAM are at a higher risk of depression, anxiety, substance abuse, smoking, bullying, and psychosocial problems. It may be necessary to provide health education and psychological counselling for adolescent girls experiencing poverty. Increases in BMI, weight and body fat percentage, waist circumference, and waist-hip ratio were also associated with AAM, with values of 0.19 kg/m^2^, 0.33%, 0.38 cm and 0.002 per 1 year of earlier AAM in China [65]. Improved nutritional intake can reduce the risks of obesity and T2DM in women [66, 67]. Moreover, studies have demonstrated that genetic regulation of puberty timing is linked with fatal disease susceptibility, such as breast and endometrial cancers [68, 69]. There are fewer health checkups among residents in poverty and rural areas that have experienced lower diagnosis and treatment levels at present in Yunnan. Therefore, people with cancers may be placed at higher risk of death. Regular screening for high-risk cancers should be encouraged among women in poverty and rural areas.

### Limitations

There are several limitations of this large sample survey. First, memory bias may be unavoidable in the retrospective study of AAM. Prospective investigation is ideal, but it is not thought to be feasible in our study population. A national survey from the United Kingdom [70] showed that 412/946 (43.6%) of participants had recalled exactly the same AAM in women aged 48 years as had been recorded during the medical examination at ages of 14–15 years. Overall, the recalled AAM values of 195 (20.6%) and 199 (21.0%) women were 1 year older and younger in middle age than those recorded in adolescence, respectively, suggesting that there was no systematic underreporting or overreporting of AAM in middle age. Additionally, the entrance school age and length of schooling are relatively regular (6 years old) in China. The grade attended at AAM can provide additional memory information. For example, if menarche occurs in the sixth grade, then 12 years of age can usually be extrapolated. Second, potential social desirability bias should not be ignored in self-reported studies. Participants likely tend to respond in a socially expected manner in regard to potentially sensitive personal questions, such as race, drug and sex issues. Recently, the meta-analyses pooled 29 studies demonstrated that the pooled correlations with social desirability were generally small (ranging from 0.06–0.11) [71]. The surveys of AAM may be less susceptible to social desirability bias because AAM is not a social pattern to be followed and is not considered as good or bad. Third, there is a potential selection bias of the sample size for women born before 1970 (0.3%) and after 2000 (0.4%). Although the proportions were very small, there were 3380 and 4985 women born before 1970 and after 2000, respectively, and enough samples were included for both groups. Fourth, a similar problem appeared for participants in the urban area (3.4%), which included 43,094 women. In fact, the proportions of the rural population were 82.6, 82.2, 79.0 and 73.3% in 1970, 1980, 1990, and 2000 in China, respectively. With the development of the economy, a large number of rural people flowed into cities, and the proportion of urban to rural residents rose to approximately 3:2 in 2019. However, most of those migrants are still classified as rural residents because of the policy restrictions of urban household registration of alteration. Finally, the population was only from Yunnan Province, although our survey covered 26 diverse ethnicities. In homogeneous ethnicities of other countries, some features of AAM may be heteromorphic, with environmental conditions and genetic susceptibility changes [72]. In summary, we do not believe that these factors would affect our findings. The present study provides a health warning for women born from 1965 to 2001 with a secular trend towards an earlier AAM in the Yunnan multiethnic population based on available evidence.

## Conclusions

Our study has shown a secular trend towards an earlier AAM during the twentieth century and early twenty-first century, and the plateau has not yet been reached in Yunnan Province, China. The magnitude and rate of variation were significantly affected by ethnicity, economic status, and area of residence. Early menarche age is associated with a variety of women’s health risks, especially in the context of low socioeconomic status. Considering the current AAM differences due to ethnic and socioeconomic status in Yunnan, we suggest that the health authority should flexibly adjust health care strategies in different regions to defend against potential health risks in women.

## Supplementary Information

**Additional file 1: Supplementary 1.** Secular trends in AAM among other ethnic minorities*.

## Data Availability

The data presented in this study are available on request from the corresponding author. The data are not publicly available because our data represent a part of the NFPHEP project, which will not be exposed until the end of the project.

## Abbreviations

AAM: The Age at Menarche
GDP: Gross Domestic Product
NFPHEP: National Free Preconception Health Examination Project
CI: Confidence Interval
