# Supplementary material for: A secular trend in age at menarche in Yunnan Province, China: a multiethnic population study of 1,275,000 women
Source: BMC Public Health. 2021 Oct 19;21:1890. doi: 10.1186/s12889-021-11951-x (PMC8524999; doi:10.1186/s12889-021-11951-x)
Supplement: Supplementary file 1 — Additional file 1: Supplementary 1. Secular trends in AAM among other ethnic minorities*. [file 12889_2021_11951_MOESM1_ESM.docx]

**Supplementary 1 Secular trends in AAM among other ethnic minorities***

|  |  | (AAM – years± SD (95%CI) ***** | | | | | | | *P* value |
| --- | --- | --- | --- | --- | --- | --- | --- | --- | --- |
| **Ethnic group** | Total (%)  N=41641 | Mean AAM | ＜1970s | 1970s | 1980s | 1990s | Early 2000s | Difference |  |
| Jingpo | 7482(17.968) | 13.639±1.238  (13.611-13.667) | 14.250±1.765  (13.129-15.371) | 13.827±1.330  (13.727-13.927) | 13.666±1.260  (13.625-13.707) | 13.556±1.179  (13.513-13.598) | 13.391±1.164  (13.046-13.737) | (-0.8587) (-1.7054, -0.012) | 0.0469 |
| Yao | 6746(16.200) | 13.803±1.241  (13.774-13.833) | 14.000±1.732 (12.836-15.164) | 14.213±1.417  (14.069-14.356) | 13.961±1.314 (13.906-14.016) | 13.697±1.169  (13.660-13.733) | 13.396±0.963  (13.211-13.582) | (-0.6038) (-1.2641,0.0565) | 0.0727 |
| Naxi | 6644(15.955) | 14.086±1.317  (14.054-14.118) | 13.667±0.707  (13.123-14.21) | 14.324±1.354  (14.217-14.431) | 14.106±1.314  (14.064-14.148) | 13.988±1.305  (13.934-14.042) | 14† | (0.3333) (-0.6061,1.2728) | 0.4475 |
| Tibetan | 5280(12.680) | 14.380±1.311 (14.345-14.415) | 15.500±1.195  (14.501-16.499) | 14.362±1.481  (14.181-14.542) | 14.441±1.359  (14.386-14.495) | 14.325±1.243  (14.277-14.372) | 13.867±0.915  (13.360-14.374) | 1.63(-2.56, -0.71) | 0.0014 |
| Blang | 3884(9.327) | 13.782±1.285  (13.742-13.823) | 14† | 13.889±1.422  (13.706-14.072) | 13.880±1.310 (13.815-13.945) | 13.696±1.248 (13.642-13.750) | 13.286±0.914  (12.758-13.813) | -0.71 (-2.14, -0.71) | 0.3014 |
| De’ang | 1672(4.015) | 13.749±1.168  (13.693-13.805) | 14† | 13.794±1.146  (13.569-14.019) | 13.838±1.204  (13.747-13.929) | 13.657±1.116  (13.579-13.735) | 13.444±1.509  (12.284-14.605) | (-0.5556) (-4.2241,3.113) | 0.7359 |
| Achang | 1634(3.924) | 13.644±1.159  (13.588-13.701) | 14.400±1.140 (12.984-15.816) | 14.047±1.107  (13.834-14.260) | 13.652±1.148  (13.574-13.729) | 13.578±1.170  (13.489-13.666) | 12.833±1.169  (11.606-14.060) | (-1.5667) (-3.1506,0.0172) | 0.0521 |
| Pumi | 1606(3.857) | 14.237±1.529  (14.162-14.311) | 13.857±1.464  (12.503-15.211) | 14.783±1.662  (14.541-15.024) | 14.292±1.509  (14.188-14.396) | 13.995±1.465  (13.878-14.112) | NA | (0.1379) (-0.9556,1.2313) | 0.8045 |
| Bouyei | 1545(3.710) | 13.701±1.177  (13.642-13.760) | 14.333±1.528  (10.539-18.128) | 13.894±1.213  (13.645-14.142) | 13.747±1.163  (13.657-13.837) | 13.645±1.181  (13.563-13.727) | 13.500±0.707  (7.147-19.853) | (-0.8333) (-4.6459,2.9792) | 0.5367 |
| Nu | 963(2.313) | 13.748±1.288  (13.666-13.829) | 13.700±0.823 (13.111-14.289) | 14.000±1.366 (13.736-14.264) | 13.893±1.340 (13.768-14.017) | 13.506±1.181  (13.389-13.624) | 14.500±0.707 (8.147-20.853) | (0.80) (-0.6021,2.2021) | 0.2324 |
| Mongol | 954(2.291) | 13.520±1.187  (13.445-13.595) | 14.000±2.000 (9.032-18.968) | 13.570±1.046  (13.335-13.804) | 13.529±1.114  (13.428-13.629) | 13.494±1.290  (13.366-13.622) | 12.500±0.707 (6.147-18.853) | (-1.50) (-6.3901,3.3901) | 0.401 |
| Jino | 943(2.265) | 13.091±1.387  (13.003-13.180) | 13† | 13.659±1.372  (13.357-13.960) | 13.087±1.414  (12.963-13.210) | 12.977±1.320  (12.838-13.116) | 12† | 1 | ＜0.0001 |
| Tujia | 384(0.922) | 13.651±1.180 (13.533-13.769) | 18† | 13.692±1.225  (13.197-14.187) | 13.565±1.166 (13.384-13.747) | 13.708±1.157  (13.544-13.873) | 13† | (0.016) (-0.4639,0.4959) | 0.9476 |
| Li | 255(0.612) | 13.682±1.312 (13.521-13.844) | NA | 13.778±1.281  (13.271-14.285) | 13.676±1.259  (13.436-13.916) | 13.692±1.380  (13.440-13.945) | 12.500±0.707  (6.147-18.853) | NA | NA |
| Manchu | 233(0.560) | 13.597±1.330  (13.425-13.768) | 17† | 14.000±1.279 (13.447-14.553) | 13.722±1.460 (13.452-13.991) | 13.312±1.073 (13.091-13.533) | 13† | (-0.6882) (-1.203,0.1734) | 0.0092 |
| Drung | 196(0.471) | 13.837±1.408  (13.638-14.035) | NA | 13.938±1.340  (13.223-14.652) | 14.020±1.400 (13.745-14.295) | 13.587±1.425  (13.259-13.914) | 14† | (0.0625) (-2.8817,3.0067) | 0.9645 |
| Shui | 149(0.358) | 13.987±1.268  (13.781-14.192) | NA | 14.100±0.994 (13.389-14.811) | 14.091±1.298  (13.772-14.410) | 13.861±1.282  (13.560-14.162) | NA | (-0.2389) (-1.0801,0.6023) | 0.5736 |
| Dong | 145(0.348) | 13.552±1.343 (13.331-13.772) | NA | 14.889±2.147  (13.238-16.539) | 13.786±1.128  (13.517-14.055) | 13.143±1.281  (12.820-13.465) | 12† | (-2.8889) (-8.1085,2.3308) | 0.2377 |

Abbreviation: AAM: Age at Menarche; NA: No applicable

* Other ethnic minorities that population included this survey more than 100 women.

† No applicable confidence interval because the insufficient sample size.
